# Utilization of genomic services among physicians in Kenya

**DOI:** 10.1371/journal.pone.0319364

**Published:** 2025-12-10

**Authors:** Hillary Sigilai, Elijah Ogola, Syokau Ilovi

**Affiliations:** Department of Clinical Medicine and Therapeutics, University of Nairobi, Nairobi, Kenya; Townsville University Hospital, AUSTRALIA

## Abstract

**Background:**

With the advancement of technology in genomics and mainstreaming of genomics across all specialties of medicine, physicians will be required to play a pivotal role in coordinating and providing primary genetic services.

**Objective:**

To describe the delivery of basic genetic services among physicians in Kenya and factors affecting the integration of genomic medicine into their practice.

**Materials and methods:**

This was an online, descriptive, cross-sectional study conducted among registered physicians practicing in Kenya and physicians-in-training (registrars) at the University of Nairobi, using an anonymous, self-administered questionnaire. The following domains were assessed: delivery of genetic services, attitudes and perceptions towards genetics, barriers to the delivery of genomic services, knowledge of genetics and physicians’ demographics. Simple random sampling method was used to recruit participants for the study. Descriptive analysis methods were used for data analysis. The data was summarized using frequencies and proportions.

**Results:**

The response rate was 41%, with 190 of the 464 eligible physicians completing the survey. Eighty-seven percent of respondents had not received formal training in genetics, with 80% reporting involvement in the evaluation of genetic patients. Physicians’ involvement in genetic testing and pharmacogenomics was low at 31% and 29% respectively. Sixty-four percent of the respondents graded their knowledge of genetics as moderate. Participants identified limited access to medical geneticist (80%), lack of referral guidelines (86%), high cost of genetic services (93%), and inadequate knowledge of genetics as barriers to genetic service delivery.

**Conclusion:**

Uptake of genomic service provision to patients by physicians remains low due to inadequate genetics training, limited number of specialists, in genetics and prohibitive costs of genomic testing. Mitigation of these factors is required to improve access to genetic care.

## Background and rationale

Genomic medicine is a branch of medicine that focuses on the prevention, diagnosis, and management of genetic disorders in patients and their families. This encompasses both monogenic/mendelian disorders and polygenic/complex trait/non-mendelian disorders. Virtually all diseases are influenced by the patients’ genetic architecture. It is therefore imperative that genetic services, such as genetic testing and genetic counseling, are incorporated into the routine clinical care of patients and their families. Genetic testing involves the analysis of human chromosomes, DNA, RNA, proteins, and metabolites to detect heritable disease-related genotypes, phenotypes with a view to establishing diagnosis, identifying at risk family members and predicting risk [1]. The National Society of Genetic Counsellors defines genetic counselling as the process of assisting individuals to comprehend and understand the medical, psychological, and familial implications of hereditary factors related to diseases [2]. With advancements in understanding the human genome, decreasing costs, and the widespread availability of genetic testing, clinical genetics is expected to continue gaining prominence in clinical care. Despite the clear benefits of clinical genetics in advancing human health, its uptake has remained low, with only approximately 50%−60% of physicians providing genetic testing and counseling [3–5].

The potential role of genomics and precision medicine, which involves tailoring interventions based on an individual’s genetic information, has long been recognized in clinical care. However, translation into mainstream healthcare has been hindered by a lack of coherent national policies, a limited number of well-trained genomic professionals, inadequate research infrastructure, and economic challenges [6–8]. Therefore, resolving these barriers to integrate genomics into healthcare and improve patient outcomes requires widespread system changes [9]. Initiatives such as implementation research help identify evidence-based strategies that facilitate the adoption of genomics by clinicians [10].

In Kenya, genetic testing is mainly available at large private hospitals and costs up to 1800 USD which is prohibitive in a Low-to-middle-income country. Most of the tests are outsourced internationally due to a lack of capacity locally. There are few medical geneticists, while genetic counselling does not exist as a discipline to support primary healthcare teams [11]. While accessibility to critical infrastructure such as genetic testing remains limited, their availability has started to increase. However, its applicability, availability, uptake, and quality have not been examined.

This study offers an overview of the current trends in clinical genetics service utilization from the perspective of physicians. In Kenya, a physician is a specialist in Internal Medicine (standard 3–4 year postgraduate training). Findings from this study will inform the formulation of institutional and departmental policies that will ensure effective utilization and delivery of genetic services in Kenya.

### Study Objective

To describe the delivery of basic genetic services among physicians and factors affecting the integration of genomic medicine into their practice.

## Materials and methods

### Study design

Descriptive cross-sectional study using an anonymous self-administered online questionnaire.

### Study population

This was a nationwide survey of physicians (specialists in internal medicine) in Kenya and registrars in internal medicine at the University of Nairobi, targeting 189 participants. At the time the study was conducted, there were 392 board-registered physicians and 72 registrars (physicians-in-training) at the University of Nairobi. The sample size was determined using Fisher’s formula for sample size computation. The computation was powered to answer the study’s objectives. The recruitment period ran from 7^th^ March 2024 to 29^th^ April 2024.

Inclusion criteria: Registered physicians practicing within Kenya and Internal Medicine registrars (physicians-in-training) at the University of Nairobi. Physicians and registrars who declined to give consent were excluded from the study.

The study population was proportionally stratified into two groups: Registered physicians and registrars. Simple random sampling was then used to recruit participants. Physicians’ and registrars’ identifiers were obtained from the Kenya Medical Practitioners and Dentists Council. A random seed was then set and executed to obtain appropriate numbers.

### Questionnaire design

The questionnaire was adopted with permission from a survey conducted by Carroll et al in 2012 in Canada [12]. The purpose of their survey was to assess the education needs of primary care physicians in preparation for enhancing their role in Genomic Medicine. It was divided into eight sections: Physicians’ demographics (9 questions), current practice characteristics (10 items), family history taking (3 items), attitude towards genomic medicine (8 items), awareness of and experience with genomic services (9 items), knowledge of genetics (14 items) and barriers to delivery of genomic services (9 items)).

### Ethical considerations

Ethical approval was sought and obtained from the Kenyatta National Hospital/University of Nairobi ethics review board (P859/11/2023). A written consent form in the English language was provided, describing the nature of the study, its risks, potential benefits, obligations, and the names and addresses of the investigators to be contacted.

### Statistical analysis

Demographic characteristics were analyzed and presented as frequencies and percentages. The Proportion of physicians who provide genomic services was presented as frequencies and percentages of physicians surveyed. Other answers were a mixture of 3–5-point Likert scales for knowledge, attitude, awareness, and barriers. Each response was analysed individually as either a positive or a negative attitude and then scored as an aggregate score using the original Bloom’s cutoff. There was a total of 10 questions, each with a score of 1–5, i.e., a minimum of 10 and a maximum of 50. Using Bloom’s cut-off points, attitude was categorized as positive/good >80% (40–50), neutral 60%−79% (30–39.5), and negative/poor <60% (<30 points). Knowledge domain was analysed as a proportion using the original Bloom’s cut-off [13]. A score <60% (< 27) was considered poor, > 60%−79% (27–35.5 points) moderate, and >80% (>36 points) was considered good knowledge.

The outcome variable ‘utilization of genomic services’ was defined as whether one had ordered carrier testing on parents of a child confirmed to have a genetic disorder, and/or ordered a genetic test for a genetic disorder. Factors associated with physicians’ use of genomic services, including training in genetics, specialty, and number of years in practice, were analysed using bivariate and multivariate logistic regression. Associations were reported by use of odds ratios and respective 95% confidence intervals. P-values of <0.05 were considered significant.

## Study results

### Baseline characteristics

The study recruited 190 participants drawn from most of the sub-specialties in internal medicine. The Majority (63%) had been in clinical practice for less than 10 years. Most respondents (87%) had not received formal training in genetics (Table 1).

**Table 1:** Demographic characteristics of the participants.

Variable	N = 190
**Sex**	
Female	109 (57%)
Male	81 (43%)
**Years in clinical practice**	
0-9	120(63%)
10-19	56 (29%)
20-29	8 (4.2%)
30-39	6 (3.2%)
**Specialty**	
Cardiology	9 (4.7%)
Dermatology	8 (4.2%)
Endocrinology	9 (4.7%)
Gastroenterology.	1 (0.5%)
Infectious disease	2 (1.1%)
Internal medicine (no sub-specialization)	79 (42%)
Nephrology	6 (3.2%)
Pulmonology	2 (1.1%)
Registrar in internal medicine	73 (38%)
Rheumatology	1 (0.5%)
**Has training in genetics** (apart from embryology)	25 (13%)
**Has a special interest in genetics**	60 (32%)

### Clinical practice of genetics

Eighty (80%) percent reported that evaluation and diagnosis of patients with genetic disorders was part of their practice, with 32% having ordered a genetic test and 24% having referred a patient for specialist genetic evaluation. Only 14 (7%) of respondents had performed carrier testing on the parents of a child with a confirmed genetic disorder.

The Majority (58%) of respondents completed a family history in over 75% of their patient populations, but only 48% updated the family history during subsequent visits. Completion of a two or three-generation family history was undertaken by 55% of the respondents (Table 2)

**Table 2:** Family History taking.

Variable	N = 190
**Proportion of patients for whom you complete a family history**	
0	2 (1.1%)
25%	33 (17%)
50%	45 (24%)
75%	69 (36%)
100%	41 (22%)
**How often is family history updated**	
At every visit	20 (11%)
Every 2–4 years	13 (6.8%)
Every 5–10 years	17 (8.9%)
Never	99 (52%)
Yearly or at periodic exams	41 (22%)
**A two or three-generation family history**	104 (55%)
**The family’s ethnic background**	116 (61%)

### Attitudes towards genomic medicine

The respondents’ attitudes towards genomic medicine were scored on a Likert scale ranging from strongly disagree to strongly agree. The responses were further categorized into three groups of negative attitude (strongly disagree and disagree), neutral attitude (neutral), and positive attitude (strongly agree and agree).

The physicians surveyed in this study had an overall positive attitude towards genomics at 60% as illustrated below (Table 3).

**Table 3:** Physicians’ attitudes towards and knowledge of genomic medicine in categories.

Category and scores (Bloom’s cut-off)	n	%	Mean (SD)
Attitude			2.4 (0.4)
Positive Attitude (80% +)	113	60%	
Neutral (60–79%)	60	31%	
Negative Attitude (< 60%)	17	19%	
Knowledge			1.7 (0.45)
High level (80% +)	113	7%	
Moderate level (60–79%)	121	64%	
Low level (<60%)	56	29%	

The majority agreed that there is a need to incorporate genomic medicine into their practice in order to improve patient outcomes. However, only 37% felt that genomics is an exciting part of their practice. Furthermore, the majority agreed that genomic medicine will make an important contribution in the management of prenatal, paediatric, and adult conditions.

### Knowledge of genetic disorders

Overall, most respondents (64%) graded their knowledge of various aspects of genetics as moderate, with only 7% having high-level knowledge as illustrated in Table 3 above.

Knowledge of emerging areas of genomic medicine, such as newer technologies entering clinical practice (low-68%), interpreting results of ‘direct to consumer’ tests (low-71%), was noticeably low. The Majority (76%) do not know how to contact their local genetics centre or find information about genetic tests within the health system (61%).

### Barriers to the provision of genetic services

Physicians identified limited access to medical geneticists (80%), a lack of referral guidelines (86%), the high cost of genomic services (93%), inadequate knowledge of genetics, and limited access to genetic testing services as barriers to the provision of genomic services as illustrated in Table 4 below.

**Table 4:** Barriers to the provision of genomic services.

Variable	N = 190^1^
**Limited access to a medical geneticist for referrals for testing and/or consultation**	
Usually	152 (80%)
**Lack of referral guidelines**	
Usually	163 (86%)
**High cost of genetic services, such as testing.**	
Usually	177 (93%)
**Inadequate knowledge of genetics, genetic testing, and genetic counselling.**	
Usually	117 (62%)
**Lack of patient interest in genetic evaluation.**	
Usually	78 (41%)
**Limited access to genetic testing services**	
Usually	155 (82%)
**Lack of detailed or updated family history**	
Usually	88 (46%)

### Correlation between physician characteristics and their utilization of genetic services

A weak positive correlation was found between the years in clinical practice and the outcome variables above (p-value 0.018). Respondents who had training in genetics were also more likely to have ordered carrier testing on parents of a child who was confirmed to have a genetic disorder or ordered a genetic test for a genetic disorder (p-value 0.037).

Years in clinical practice and whether one had received training were fit into the multivariate model. It was found that doctors with more than 9 years of clinical practice were more likely to utilize genomic services compared to those with less than 9 years (adjusted OR = 1.16, 95% CI [1.16–4.09], p = 0.016). Doctors who received training in genetics were more than twice as likely to utilize genetic services compared to those who did not (aOR = 2.60, 95% CI [1.09–6.29], p = 0.031).

## Discussion

This study aimed to provide the first comprehensive view of physicians’ involvement in genomic medicine, their awareness of currently available genetic services, attitudes towards genomic medicine, and barriers to integrating genomic medicine into mainstream healthcare in Kenya.

In this study the majority of participating physicians reported that they undertake the clinical aspects of genomic medicine, including the evaluation and diagnosis of patients with genetic disorders, referral of patients for specialised genetic services, and provision of education about genetic conditions. This suggests that basic genetic principles are integrated into medical practice. Similar findings have been reported in other studies, including a Canadian survey of family physicians, by Suchard et al and Hayflick et al [5,14,15]. However, involvement in areas such as genetic counselling and pharmacogenomics is low. In the absence of certified genetic counsellors in Kenya, the role of physicians in counselling individuals with genetic disorders remains integral. Similarly, Haga et al found that only 13% of respondents felt comfortable ordering pharmacogenomic tests, while a quarter reported not having any education about pharmacogenetics [16].

Worryingly, 68% of physicians have never ordered a genetic test for a genetic disorder. In addition, majority reported that they do not discuss benefits, risks, or limitations of genetic tests with patients or provide support to patients coping with genetic results. Even fewer know where to refer patients for genetic evaluation. The low uptake of genetic testing in our setting could be due to a lack of training relevant to genetic testing, as well as a lack of knowledge and experience and limited testing infrastructure. The literature is mixed in this regard. In a 2008 survey of primary care physicians in the US, 60% of physicians had ordered a genetic test [3]. Genetic testing has the potential to decrease morbidity and mortality, mainly when used for risk profiling early in the management of chronic diseases [3].

Family history is a fundamental tool in clinical genetics, key to risk assessment and identifying individuals who may benefit from further genetic evaluation [17]. An essential aspect of family history taking is the collection of adequate amounts of information and its proper interpretation. Fifty-eight percent (58%) of physicians reported that they collect family history in 75%−100% of patients seen, with the majority incorporating two or three generations (55%), the family’s ethnic background (61%) and medical risk factors (93%). Previous surveys among physicians have demonstrated an imbalance between the frequency and quality of family history taking [14,18]. The development of standardized family history-taking tools, including checklists, with an emphasis on proper history taking as part of medical education, can be used to build on existing knowledge and skills in eliciting family history.

The study also investigated physicians’ attitudes towards genomic medicine. Overall, 60% of physicians surveyed in this study had a positive attitude towards genomic medicine. More than two-thirds of respondents felt that there is a need to incorporate genomic medicine into their practice and keep up to date with advances in genomic medicine. Over half of the responding physicians agreed there are sufficient benefits to warrant testing for inherited adult-onset diseases and were convinced advances in genomic medicine will improve patients’ outcomes. This can be leveraged to enhance the integration of genomic medicine into mainstream healthcare. However, the majority do not find genomics an essential part of their practice. In a South African study examining knowledge and attitudes towards predictive testing, attitude was largely positive despite the costs, as respondents perceived benefits outweighed costs [19]. The results of this study also support existing literature where participants felt that genomic medicine is likely to have an impact on clinical practice in the future [4,14].

Knowledge of genetics has been found to translate into increased adoption of genomic medicine in mainstream healthcare [19]. Most (64%) of the respondents rated their overall knowledge in genomic medicine as moderate, i.e., 60–79% on Bloom’s cutoff. Knowledge in basic concepts such as genetic consequences of consanguinity and genetic risk factors of common complex disorders was rated as moderate to high. However, knowledge of advanced/emerging themes such as the interpretation of direct-to-consumer testing and pharmacogenomics, was largely poor. Specific educational interventions should be geared towards these areas. Self-perceived knowledge deficits are a global problem [20]. More data is needed to determine specific educational resource needs and develop genetic educational programmes targeting physicians.

Systemic and individual barriers to the provision of genetic services have been identified in literature [6,11,21]. In this study physicians identified limited access to medical geneticists (80%), lack of referral guidelines (86%), high cost of genetic services (93%), inadequate knowledge of genetics, among others. These barriers impact practice, further impeding the delivery of care to patients in need of genetic services. Based on these findings, multiple strategies can be deployed to ameliorate access to genetic services.

### Study limitations

The main limitation of the study was the low response rate. Despite attaining the desired sample size of 189, the overall response rate was low due to factors such as the length of the questionnaire and inertia among potential study participants. The possibility of response bias due to self-reporting is also noted. This was addressed by wording questions neutrally and ensuring answer options were open-ended. In addition, a mixed qualitative/quantitative study using one-on-one interviews would have provided more valuable information on the state of genomic medicine in Kenya, which is essential for designing effective implementation strategies for incorporating it into mainstream healthcare.

The study also lacked statistical power to analyse the secondary objective.

## Conclusion and recommendations

Our study offers valuable insights into the current state of genomic medicine in Kenya. Basic principles of genetics, such as the evaluation and diagnosis of genetic conditions, the referral of patients for specialised genetic services, are incorporated into medical practice. However, physicians’ uptake of genetic testing and counselling is low, and their knowledge of genomic medicine is inadequate. Based on our findings, successful implementation of genomic clinical care pathways will require:

i Development of genetics training programmes with a focus on family history taking, genetic counselling, and genetic testing modalities.
ii Investment in infrastructure, such as genomic testing laboratories and training of personnel, including medical geneticists and genetic counsellors.

### Disclosures

This work was completed by H.S. in part fulfilment of Master of Medicine in Internal Medicine from the University of Nairobi.

No artificial intelligence tools were used in the production of this work.

## Supporting information

S1 File Final results; utilisation of genomic services results. (DOCX)
