# Supplementary material for: Utilization of genomic services among physicians in Kenya
Source: PLoS One. 2025 Dec 10;20(12):e0319364. doi: 10.1371/journal.pone.0319364 (PMC12694833; doi:10.1371/journal.pone.0319364)
Supplement: S1 File — (DOCX) [file pone.0319364.s001.docx]

##
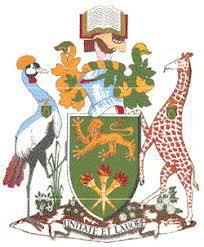


##

**UTILISATION OF GENOMIC SERVICES AMONG**

**PHYSICIANS IN KENYA.**

**DR. SIGILAI KIBET HILLARY**

**H58/40563/2021**

**A thesis submitted in partial fulfilment for the award of the degree of Master of Medicine in Internal Medicine,**

**University of Nairobi**

**September,2024**

# STUDENT’S DECLARATION,

I, **Dr.** **Sigilai Kibet Hillary,** do hereby declare that this is my original work and that it has not been presented before for a degree or any other academic award at this or any other university.

**Dr. Sigilai Kibet Hillary (MBCHB- UON)**

**H58/40563/2021**

**.**

Signed………………………………………. Date:………………………………..

# SUPERVISORS’ APPROVAL

This thesis has been submitted with our approval as the supervisors:

**Professor E.N Ogola,**

Consultant cardiologist, Professor of Medicine, Department of Clinical Medicine and Therapeutics.

University of Nairobi

Signed………………………………………. Date:………………………………..

**Dr. Syokau Ilovi**

Consultant Physician, Medical Geneticist, Department of Clinical Medicine and Therapeutics.

University of Nairobi

Signed………………………………………. Date:………………………………..

# TABLE OF CONTENTS

[i](#_Toc171887089)

[STUDENT’S DECLARATION, ii](#_Toc171887090)

[SUPERVISORS’ APPROVAL iii](#_Toc171887091)

[TABLE OF CONTENTS iv](#_Toc171887092)

[LIST OF TABLES vi](#_Toc171887093)

[LIST OF ABBREVIATIONS viii](#_Toc171887094)

[OPERATIONAL DEFINITIONS ix](#_Toc171887095)

[ABSTRACT x](#_Toc171887096)

[1.0 CHAPTER ONE. 1](#_Toc171887097)

[INTRODUCTION 1](#_Toc171887098)

[1.1 Background Information 1](#_Toc171887099)

[2.0 CHAPTER TWO. 3](#_Toc171887100)

[LITERATURE REVIEW 3](#_Toc171887101)

[2.1 Definitions 3](#_Toc171887102)

[2.2 Burden of Genetic Diseases 3](#_Toc171887103)

[2.3 Integration of Clinical Genomics into Mainstream Healthcare. 4](#_Toc171887104)

[2.4 Provision of Genetic Services at Primary Level. 5](#_Toc171887105)

[2.4.1 Genetic Testing 7](#_Toc171887106)

[2.4.2 Genetic Counselling 8](#_Toc171887107)

[2.4.3 Family History Taking 8](#_Toc171887108)

[2.5 Specialized Genetic Services 8](#_Toc171887109)

[2.6 Barriers to Provision of Genetic Services 10](#_Toc171887110)

[2.7 Problem Statement 10](#_Toc171887111)

[2.8 Study Justification 11](#_Toc171887112)

[2.9 Research Question 12](#_Toc171887113)

[2.10 Study Objectives 12](#_Toc171887114)

[2.10.1 Broad Objective 12](#_Toc171887115)

[2.10.2 Specific Objectives 12](#_Toc171887116)

[2.10.2.1 Primary Objectives 12](#_Toc171887117)

[2.10.2.2 Secondary Objective 12](#_Toc171887118)

[3.0 CHAPTER THREE 13](#_Toc171887119)

[STUDY METHODOLOGY 13](#_Toc171887120)

[3.1 Study Design 13](#_Toc171887121)

[3.2 Study Setting 13](#_Toc171887122)

[3.3 Study Population 13](#_Toc171887123)

[3.4 Inclusion and Exclusion Criteria 13](#_Toc171887124)

[3.4.1 Inclusion Criteria 13](#_Toc171887125)

[3.4.2 Exclusion Criteria 13](#_Toc171887126)

[3.5 Sample Size Calculation 13](#_Toc171887127)

[3.6 Sampling Technique 14](#_Toc171887128)

[3.7 Data Collection 15](#_Toc171887129)

[3.8 Study instrumentation 15](#_Toc171887130)

[3.8 Study Variables 16](#_Toc171887131)

[3.9 Quality Assurance 16](#_Toc171887132)

[3.10 Data Management and Analysis 17](#_Toc171887133)

[3.11 Ethical considerations. 17](#_Toc171887134)

[CHAPTER FOUR. 19](#_Toc171887135)

[RESULTS 19](#_Toc171887136)

[4.1 Characteristics of the study participants. 19](#_Toc171887137)

[4.2 Current Practice characteristics. 21](#_Toc171887138)

[4.3 Attitudes towards Genomic Medicine 25](#_Toc171887139)

[4.4 Knowledge of genetic disorders and awareness of genetic services. 28](#_Toc171887140)

[4.4.1 Knowledge of Genetics 28](#_Toc171887141)

[4.4.2. Awareness of genetic services. 30](#_Toc171887142)

[4.5. Barriers to the provision of genetic services 31](#_Toc171887143)

[4.6 Correlation between physician characteristics and their utilization of genetic services. 32](#_Toc171887144)

[CHAPTER FIVE 35](#_Toc171887145)

[5.1 Discussion 35](#_Toc171887146)

[5.2 Conclusion 37](#_Toc171887147)

[5.3 Recommendations 37](#_Toc171887148)

[5.4 Study strengths 38](#_Toc171887149)

[5.5 Study Limitations 38](#_Toc171887150)

[5.6 Study Significance and Results Dissemination 38](#_Toc171887151)

[REFERENCES 39](#_Toc171887152)

[APPENDICES 43](#_Toc171887153)

[Appendix A: Participants Consent 43](#_Toc171887154)

[Appendix B: Questionnaire 46](#_Toc171887155)

[Appendix C: Permission For Use of Questionnaire. 53](#_Toc171887156)

[Carroll, Dr. June <June.Carroll@sinaihealth.ca> 53](#_Toc171887157)

# LIST OF TABLES

[Table 1: Common conditions with genetically predisposed subgroups. 3](#_Toc171551848)

[Table 2: Family History taking 20](#_Toc171551849)

[Table 3: Overall Attitude towards Genomic medicine 21](#_Toc171551850)

[Table 4: Physicians attitudes towards genomic medicine in categories 21](#_Toc171551851)

[Table 5: Overall knowledge of genetics 24](#_Toc171551852)

[Table 6:Physicians' Self-perceived knowledge of genetics in categories 24](#_Toc171551853)

[Table 7: Awareness of Genetic services. 26](#_Toc171551854)

[Table 8: Barriers to the provision of genetic services 27](#_Toc171551855)

[Table 9: Correlation between selected physician characteristics and outcome variables. 29](#_Toc171551856)

**LIST OF FIGURES**

[Figure 1: Diagram showing the role of primary care giver in provision of genetic services in bold.(18) 5](#_Toc171552048)

[Figure 2: Current practice characteristics 18](#_Toc171552049)

[Figure 3:Proportion of physicians who have referred a patient for genetic evaluation. 19](#_Toc171552050)

[Figure 4: Ordered Carrier testing 19](#_Toc171552051)

# LIST OF ABBREVIATIONS

**ADPKD-**  Autosomal Dominant Polycystic Kidney Disease.

**APC-** Adenomatous polyposis Coli

**BRCA-** Breast Cancer Gene

**CHD-** coronary heart disease

**CME-** Continuous Medical Education

**DNA-** Deoxyribonucleic Acid

**ECSACOP-** East, Central and Southern Africa College of Physicians.

**KMPDC-** Kenya Medical Practitioners and Dentists’

**LDL-** Low Density Lipoproteins

**MSH2-** MutS humolog 2

**PCP-** Primary Care Physicians.

**RNA-** Ribonucleic Acid.

**SCD-** Sudden Cardiac death

**UON-** University of Nairobi

# OPERATIONAL DEFINITIONS

**Physician:** A certified specialist trained in Internal Medicine. Internal medicine is a specialty involving the prevention, diagnosis, and treatment of adult disease conditions. Sub-specialties include nephrology, neurology, cardiology, gastroenterology, endocrinology, rheumatology, infectious diseases, critical care medicine, geriatric medicine, pulmonology and haemato-oncology.

**Registrar:** Doctors undergoing post-graduate training in internal medicine.

**Attitude:** An emotional state or a thought process that shapes an individual’s conduct. It encompasses positive or negative sentiments towards a specific subject, individual, or activity. Individual’s attitudes or perceptions can positively or negatively influence their decision-making process.

**Genomic Medicine:** Branch of medicine that involves the use of a patient’s genotypic information in his or her care. It Facilitates individually tailored preventive, diagnostic and therapeutic regimens.

**Genetic services**: Defined as Patient-centered activities that include screening for treatment and prevention, counselling regarding genetic testing results, referring patients with genetic risks, and comprehensive family history-taking.

**Basic genetic services**: Genetic services that can be appropriately delivered by individuals without specialist training in genetics. These include diagnosis of common genetic disorders from history taking, working with geneticists to monitor health of individuals with genetic disorders, basic genetic education of patients and families, organizing care for patients with complex genetic service requirements through appropriate referral to genetic specialists.

**Genetic specialist**: A medical doctor or healthcare worker with specialized training in genetics. They include medical geneticists and genetic counsellors.

#

# ABSTRACT

**Background:** Physicians are key in the provision of genetic services for common conditions such as breast cancer, cardiovascular disease, Alzheimer disease and sickle cell disease. Genetic services are delivered at the primary level by providers such as physicians or at specialty level by medical geneticists and genetic counsellors. Currently there are only two registered medical geneticist and no trained genetic counsellors in Kenya. Consequently, physicians will be required to play a pivotal role in the coordination and provision of genetic services. Availability of critical infrastructure such as genetic testing remains limited, but their availability has started to increase. However, their applicability, availability, uptake, and quality has not been examined.

**Objectives:** To describe the delivery of basic genetic services among physicians and factors affecting integration of genomic medicine into their practice.

**Materials and Methods:** This was an online descriptive cross-sectional study conducted among physicians and registrars registered by the Kenya Medical Practitioners and Dentists Council. Simple random sampling method was used to recruit participants into the study. Interview of participants was conducted using an online close-ended questionnaire. The following domains were assessed: Delivery of genetic services by physicians, attitude and perceptions towards genetics, barriers to delivery of genomic services, knowledge of genetics and physician demographics/characteristics. Descriptive analysis methods were used for data analysis. The data was summarized using frequencies and proportions.

**Results:** Four hundred and sixty-four (464) physicians were eligible for the survey. The final response rate was 41% (190/464). Eighty percent (80%) of respondents are involved evaluation and diagnosis of patients, 63% routinely offer referrals for genetic services. Involvement in genetic testing, pharmacogenomics and genetic testing was low at 31%, 29% and 25% respectively. One hundred and thirteen respondents (60%) recorded a positive attitude towards genomic medicine, while majority (64%) graded their knowledge of genetics as moderate. Participants identified limited access to medical geneticist (80%), lack of referral guidelines (86%), high cost of genetic services (93%), inadequate knowledge of genetics as barriers to genetic service delivery.

**Conclusion:** Evaluation and diagnosis of patients with genetic conditions and referral of patients for genetic evaluation were part of majority of respondents’ practice. However, there is low uptake of genetic testing, genetic counselling and pharmacogenomics. The physicians had a positive attitude towards genomic medicine. Barriers to access/provision of genetic services were limited access to medical geneticists for referral, high cost of genetic services such as testing, inadequate knowledge of genetics and limited access to genetic services. Despite progress, challenges remain in integration of genomic medicine into clinical practice.

#

# CHAPTER ONE.

# INTRODUCTION

## 1.1 Background Information

Genomic Medicine is a branch of medicine that involves the use of a patient’s genotypic information in his or her care(1). This definition encompasses both single (Mendelian) and complex multigenic diseases. It is expected to improve the quality of healthcare by providing insight to etiology of disease and facilitating individually tailored prevention and treatment regimens(2).

Management of heritable diseases, illnesses and syndromes relies heavily on the delivery of genetic services. The global burden of genetic disorders up to the age of 25 years is estimated at 5.3%(3). Annually, up to 7.9 million newborns have genetic or congenital disorders with majority of these found in low and middle income countries (4).

The human genome project which involved sequencing of the whole human genome was completed in 2003 and heralded the beginning of the Genomic era of medicine. This has resulted in a paradigm shift in the practice of medicine, with genomics used in identification of new pathogens. Pharmacogenetics is used in the design and implementation of new drug therapies while the genetic component of common conditions such as hypertension, diabetes and asthma has been elucidated (5).

Despite the clear potential of these advancements, substantial hurdles for actual implementation and delivery of genetic services remain. Between 2010 and 2018, several studies carried out in high-income countries examined utilization and uptake rates, which varied greatly and were impacted by patient preference, provider knowledge, and aspects of the health system. For example, BRCA testing rates for Hereditary Breast and Ovarian Cancer (HBOC) stood at 61% in the United States with a 90% uptake in genetic counselling among this group of patients. There was, however, a sustained increase in uptake of genetic services across the same timeframe (6)(7).

In Kenya provision of clinical genetic services remains limited, but it’s availability is increasing. This study examined their uptake, quality, and accessibility from the viewpoint of medical practitioners. We also attempted to elucidate barriers and facilitators to the organization and implementation of these services into mainstream healthcare delivery.

# 2.0 CHAPTER TWO.

# LITERATURE REVIEW

## 2.1 Definitions

Genetic services can be defined as patient-centered activities that include screening for, treatment and prevention, counselling regarding genetic testing results, referring patients with genetic risks and comprehensive family history taking(6). The treatment and prevention of illnesses where a substantial genetic component contributes to their cause is the aim of genetic health interventions. They also help to guide precision medicine, shorten time taken to make a diagnosis and initiate screenings based on familial risk (8).

## 2.2 Burden of Genetic Diseases

Virtually all diseases have genetic component and an environmental component. Disease is the result of environmental-gene interactions. While chromosomal abnormalities and monogenic disorders affect less than 2 % of the population, most diseases have a multifactorial component. As genetic factors in common diseases are identified and gene-based therapies are developed, modern geneticist will play crucial role in the care of an increasing number of patients.

Single gene defects include autosomal dominant and recessive disorders, X-linked disorders and Y-linked disorders. Collectively they comprise approximately 0.4 % of all live births resulting in substantial morbidity well into adulthood (9). In Kenya Sickle cell disease is the most common inherited disorder in the Western part of the country with about 4.5% of children born with SCD and 18% of children born with sickle cell trait(10).

While single gene and chromosomal disorders are individually rare, some common conditions also have strong underlying genetic components as illustrated in the table below.

Table 1: Common conditions with genetically predisposed subgroups.

| **Condition** | **Sub-group with genetic pre-disposition** | **Genetic Variation** |  |
| --- | --- | --- | --- |
| Breast Cancer | About 5%of breast Cancers occur in women with BRCA1 or BRCA2 mutation | BRCA1 and BRCA2 mutation |  |
| Diabetes | Maturity-onset diabetes in young people. | Mutation in MODY gene |  |
| Arrhythmia/SCD | Ion channelopathies | Multiple genes pre-dispose to arrhythmias and SCD |  |
| Hypercholesterolemia/ premature CHD | Familial hypercholesterolemia | LDL receptor gene or apolipoprotein B gene |  |
| Bowel Cancer | ~ 5% are due to inheritance of genes associated with lynch syndrome. | -APC gene related familial polyposis.  -MSH2, mih1, myh genes |  |
| Obesity | Severe early onset obesity | Genes involved in production of leptin. |  |

##

Genetic diseases and genetic factors of diseases play a major role in development of adult-onset non-communicable diseases. However a considerable proportion of physicians fail to recognize the significance of genetics in their clinical practice, believing that genetic disorders are uncommon and incurable(11).

## 2.3 Integration of Clinical Genomics into Mainstream Healthcare.

The double helix structure of DNA was discovered in 1953 giving rise to modern genetics and yielding important insights into how genes control chemical processes within cells(12). Subsequently, the genetic code was deciphered in 1961 with Sanger sequencing (sequencing by synthesis) developed in 1977. Sanger sequencing resulted in completion of various genome sequences and provided a foundation for the development of other sequencing techniques collectively known as Next Generation Sequencing (NGS)(13). Genomic sequencing enables the correlation of genotypes with phenotypic characters.

The completion of the human Genome Project in 2003 resulted in an exponential growth in our understanding of the genetic basis of many diseases and the creation of new strategies for their prevention and treatment(14). It provided basic raw DNA sequence that gave rise to a plethora of secondary studies that which improved our knowledge of structure and functioning of the genome (15). Healthcare is expected to change because of the widespread use of genomic technology to give patients diagnostic, prognostic, and therapeutic information.

The United Kingdom which is a considered a global leader in genetics and genomics, released its strategic plan to enable the provision of genomic healthcare to its population centered around 3 pillars: diagnosis and personalized medicine, prevention and early detection and research (16). Despite these achievements integration of genomics into healthcare has been slow because of the scarcity in access to genomic services across most specialties.

As a result of genomics research into highly common complex illnesses like obesity, diabetes, and asthma, doctors will play a bigger role as primary level genetic service providers. They will be counted on to guide patients through the options for testing and treatment. As such, they need to be knowledgeable about the genetic underpinnings of many genetically influenced diseases, be able to assess risk for a particular mutation based on personal and family history and be qualified to offer counselling (17).

## 2.4 Provision of Genetic Services at Primary Level.

Some basic genetic services are delivered appropriately by physicians. These Primary level genetic services include.

1. Identification of people with genetic disorders and those at risk of developing or transmitting one.
2. Diagnosis of common genetic disorders from history taking and physical examination.
3. Working with genetic specialist to monitor the health of individuals with genetic disorders.
4. Basic genetic education of patients and families.
5. Organizing care for patients with complex genetic service requirements.
6. Facilitating use of genetic services.
7. Appropriate referral to genetic specialists for testing and counselling(18)(17).


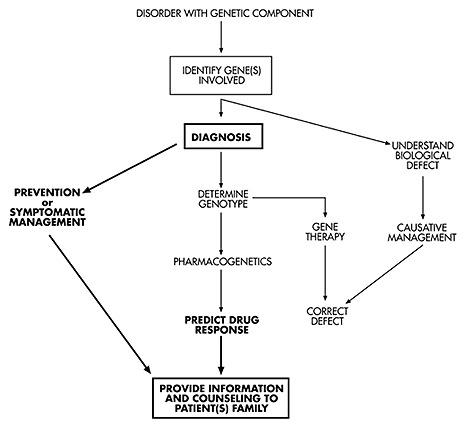


Figure 1: Diagram showing the role of primary care giver in provision of genetic services in bold.(18)

### 2.4.1 Genetic Testing

Genetic testing involves the analysis of human DNA, RNA, proteins, and certain metabolites to detect heritable disease-related genotypes, mutations, phenotypes or karyotypes for clinical purposes. Genetic testing can be used to establish prenatal and clinical diagnosis or prognosis, identify carriers, and predict disease risk. Included are tests in high risk families as well as prenatal, newborn and carrier screening (19).

Genetic Testing technologies currently available include Whole Exome Sequencing, Whole Genome sequencing, targeted next generation sequencing and Sanger sequencing. In Kenya testing is mainly available at the large referral hospitals and private laboratories and costs between Kenya Shillings 5000-240000. Some of the tests are outsourced internationally due to lack of capacity locally.

### 2.4.2 Genetic Counselling

The National Society of Genetic Counselors in the United States defined Genetic Counselling as the process of helping people understand and adapt to the medical, psychological, and familial implications of genetic contributions to disease. This process involves the following.

1. Analyzing medical or family history to determine the likelihood of a disease developing or recurring.
2. Providing information regarding inheritance, testing, management, prevention, genomic resources, and research
3. Counselling to promote informed choices and adaptation to the risk or condition. (20)

Studies on BRCA 1/2 testing including a systematic review demonstrated that genetic counselling is associated with reduced anxiety, worry and depression and enabled increased understanding of risk among patients and their family members (21).

### 2.4.3 Family History Taking

Family history taking on the other hand is the most useful tool for risk assessment for common disorders available to physicians. The probability of developing various diseases such as stroke, type 2 diabetes and cardiovascular disease is increased for people with an affected first degree relative (22). A basic family history should include three generations and contain the following information:

1. Baseline information such as names and dates of birth.
2. Racial/ethnic background of the family.
3. Health status including medical conditions and age at diagnoses.
4. Cause of death of each family member and the age at death.
5. Pregnancy outcomes of patient and genetically related relatives.

There are various ways to document a family history, such as pedigrees, forms, checklists, and charts. The best way to gather information about family history is through pedigrees (23).

## 2.5 Specialized Genetic Services

Genetic specialists on the other hand assess genetic risk and coordinate testing. They possess sophisticated knowledge in genetics which primary care physicians may lack and are formally trained and certified in genetics ( genetic counsellors , medical geneticists, genetic nurses)(24). There are 2 registered medical geneticists in Kenya while genetic counselling does not exist as a discipline to support the primary healthcare teams (25). Therefore, there is need for capacity building in these areas to improve service availability and accessibility.

Prompt and appropriate referral for specialist genetic services is crucial to prevent misinterpretation of genetic results, provision of incorrect information to patients and unnecessary treatment procedures. Referral patterns can be predicted by physician’s demographics such as specialty, gender, year of graduation and availability of educational resources (18). Physicians with higher genetics knowledge and those who practice in academic settings were found to be more likely to refer their patients to genetic services (26).

The quality of genetic service delivery is variable in low and middle income countries (5). In Kenya, studies carried out among specific patient populations reveal that patients have limited access to genetic testing and counselling services. In a qualitative study of 21 breast cancer survivors conducted via focus groups most participants reported limited experience with counselling about genetic risk and expressed the need for more comprehensive counselling and genetic testing (27). Genetic services are however becoming increasingly available for a wide variety of conditions, but their uptake, accessibility and availability has not been properly investigated.

A few studies have assessed physicians’ use of genetic services. A national survey of US Primary Care Physicians in 2008 showed that 60% of Primary care physicians have ordered a genetic test and 74% referred a patient for genetic evaluation (28). In Texas (2016), out of 157 PCPs surveyed only half reported that they were aware of genetic testing services in their area despite the increasing relevance of genomics in their practice (29). Use appears to vary by specialty. In Another survey of 401 internists carried out by Hayflick et al(18) in 1998 in the pacific northwest using various clinical scenarios; Less than half (50%) routinely sought specialist genetic services for genetic conditions, 54-80% completed a two or three generation family pedigree depending on the clinical scenario offered, with >75% of those interviewed providing risk counselling and genetic testing. In South Africa, 50.9% of allied health professionals surveyed knew that genetic counselling services were available but only 51.7% of those who knew of the service had referred patients to these services in the preceding year indicating underutilization of genetic counselling services (30).

Overall genomic medicine has been adopted to some extent in neurology, cardiology and oncology (31)(32)(33). Around 87% of physicians were aware of BRCA testing in patients with Breast Cancer with 25 % having ordered a test in the past year in an online survey conducted among physicians in 2007 (34).

## 2.6 Barriers to Provision of Genetic Services

Physicians face many challenges in accessing and delivering genetic services, with most centered around limitations in finance, human and technical resources. Physicians have identified some barriers to the access and provision of genetic services in routine practice. These include lack of confidence in genetics knowledge, largely as a result of having little experience or training relevant to genetics.(35)(36); Lack of detailed family history taking -with the aim of obtaining a pedigree that includes at least 3 generations(37), lack of awareness of genetic services, lack of referral guidelines or tools to facilitate their use.

Others barriers to the provision of genetic services include high cost of genetic services, insurance limitations, the impression of minimal or no benefit from genetic services, sceptism about clinical utility of genetic testing and the lack of information on how to manage moderate risk for genetics related disease (6).

Locally, a qualitative study was conducted among providers of genetic services to explain and examine the current landscape of genetic service delivery. Despite the limited number of participants, the study exposed several challenges facing genetic service delivery in Kenya key among them: limited knowledge of genetics, high cost of genetic testing, and counselling challenges. Potential opportunities identified include increased awareness among the general public leading to more demand for tests as well as an increase in medical utility of genetic testing (38).

In the absence of genetic specialists, physicians play a critical role in delivering genetics-related services to adults with genetic diseases. This study will be the first in Kenya to generate quantitative data regarding the extent to which physicians deliver basic genetic services in their practice, their awareness of these services, and attitudes towards genomic medicine.

## 2.7 Problem Statement

Genomic service such as testing, counselling and risk assessment are essential for the proper management of heritable illnesses, diseases, and syndromes. As mentioned earlier, the burden of these disorders is high especially in low and middle income countries such as Kenya (3).

Despite increasing availability of genetic services, uptake remains low as documented in several studies. This study focused on physicians’ awareness of genetic testing and services, their competency in skills related to provision of genetic services as well as barriers to the effective delivery of genomic services. Findings from this study will be crucial in the development of an evidence-based model for genetic service delivery in Kenya.

## 2.8 Study Justification

Addressing barriers to implementation of genomic services is complex and requires a multilevel approach that targets patients, provider, organizational and policy issues. It is important to improve our understanding of these barriers so that strategies can be developed and implemented to improve integration of genetic services into healthcare delivery. Genetic diseases and genetic factors of diseases play a major role in development of adult onset non-communicable diseases (39). However a considerable proportion of physicians fail to recognize the significance of genetics in their clinical practice, believing that genetic disorders are uncommon and incurable (11).

Advances in genetics mean than genetic services such as genetic testing are available for a variety of conditions. The growth of institutions offering these services will force Kenyan physicians to incorporate genetics into their clinical practice. Previous research shows that many physicians lack the comprehensive set of skills required to provide adequate genetic services such as knowledge of basic genetics, the ability to accurately interpret genetic results and the understanding of ethical, legal and psychological implications of genetic testing (40).

This study offers an overview of the current trends in clinical genetics service utilization in Kenya from the perspective of physicians. Findings from this study will aid in the formulation of institutional and departmental policies that will ensure effective utilization and delivery of genetic services in Kenya.

## 2.9 Research Question

What is the level of provision and utilization of currently available genetic services among physicians in Kenya?

## 2.10 Study Objectives

### 2.10.1 Broad Objective

To describe the delivery of basic genetic services among physicians and factors affecting integration of genomic medicine into their practice.

### 2.10.2 Specific Objectives

#### 2.10.2.1 Primary Objectives

1. To determine the proportion of physicians practicing in Kenya who provide basic genetic services.
2. To determine physicians’ attitude and perception towards genetics.
3. To determine physicians’ knowledge of genetics.
4. To determine the perceived barriers to genetic service delivery among physicians.

#### 2.10.2.2 Secondary Objective

To evaluate the correlation between selected characteristics of physicians and their utilization of genomic services.

# 3.0 CHAPTER THREE

# STUDY METHODOLOGY

## 3.1 Study Design

Descriptive cross-sectional study using an anonymous self -administered online questionnaire.

## 3.2 Study Setting

This was a nationwide survey targeting physicians in Kenya and Internal Medicine registrars at the University of Nairobi.

## 3.3 Study Population

Qualified Physicians actively practicing in Kenya. Currently there are 392 physicians registered by the Kenya Medical practitioners and Dentist’s Council- which is the profession’s regulatory body (41). The study also recruited Registrars (physicians in training) from the University of Nairobi.

## 3.4 Inclusion and Exclusion Criteria

### 3.4.1 Inclusion Criteria

i. Registered physicians practicing within Kenya. Registrars (physicians-in-training) at the University of Nairobi.

ii. Completion of the study questionnaire.

### 3.4.2 Exclusion Criteria

1. Physicians and registrars who declined to give consent.

## 3.5 Sample Size Calculation

The sample size was determined using the Fishers formula for sample size computation for cross-sectional studies. The computation will be powered to answer the study’s objective.

$$n=\frac{{(Z_{1-\alpha/2}+Z_{1-\beta})}^{2}P(1-P)}{{(d)}^{2}}$$

Where;

**n** = sample size

$\boldsymbol{Z}_{\boldsymbol{1-\alpha/2}}$ = Z statistic for a level of confidence, take at 95% = 1.96

$\boldsymbol{Z}_{\boldsymbol{1-\beta}}$ **=**Power at 80% = 0.84

**P** = Estimated true proportion of physicians who deliver basic genetic services, Taken at the assumed rate of 79% from a study by Truong et al .(8)

**d** = Precision of the study that will be taken at 7% based on results from ((18)

$$n=\frac{({1.96+0.84)}^{2}*0.79*(1-0.79)}{{(0.07)}^{2}}$$

n = 131

Since the population of physician and registrars are known, the study will apply finite population correction to get an adjusted sample size.

$$n_{1}=\frac{n_{0}*N}{n_{0}+(N-1)}$$

Where:

N = the expected population of physicians (392) and registrars (72) = 464

$n_{0}$ = the calculated powered sample size = 266

$$n_{1}=\frac{266*464}{\begin{aligned} 266+(464 \\ -1) \end{aligned}}$$

n_1_ = 171

Including a 10% attrition rate to cater for non-response, we will have a sample size of:

n = 171*1.1

=189

Therefore, the study will collect data from a minimum of 189 physicians and registrars.

## 3.6 Sampling Technique

The study population was proportionally stratified into two: Registered physicians and registrars (physicians in training). The stratification was proportionate to the number of physicians and registrars within the University of Nairobi**.** The first stage involved the selection of a study sample proportionate to size of the population.

Study population / Total population * Sample size

Physicians: 392/464 = 159

Registrars: 72/464 = 30

The second stage involved a simple random selection of the physicians and registrars. This was performed by listing their identifiers as obtained from KMPDC and Deans of school of medicines respectively in a statistical software (R version 4.2 or MS Excel). A random seed was set and executed to obtain the appropriate numbers of the study sample. This gave an equal chance of selection of registrars and physicians and is representative of physicians in the country.

## 3.7 Data Collection

Data was obtained and stored using an electronic tool, which was a google form using a multi-structured questionnaire. Collection of data was done by the Principal Investigator in collaboration with a study assistant (medical student) who was trained on the study protocol. The self-administered questionnaire was sent to participants via email and or social media platforms until a response was obtained. However, when no response was obtained after 3 weeks it was declared a non-response. A subsequent simple random sampling was repeated until the sample size was achieved.

## 3.8 Study instrumentation

The questionnaire was obtained with permission from a survey carried out by Carroll et al in 2012 in Canada. It was developed by a multi-disciplinary team and adapted for use in the study environment. The questionnaire was validated by a medical geneticist and cardiologist from the University of Nairobi. Content and face validation of the study questionnaire to ensure it achieves the research objectives was done by the team above. The study questionnaire was piloted among seven physicians and registrars and the feedback used to improve the rigor and conciseness of the study tool.

It describes physician demographics and characteristics (6 items). Assesses delivery of genetic services in the following domains: 1) Family history taking, 2) Referral for genetic evaluation, 3) Risk assessment, 4) Genetic counselling, 5) Genetic testing. There are questions on attitude towards genomic medicine (10 items), awareness of and experience using genetic services (11 items), self-perceived knowledge of genetics (9 items), and barriers to the delivery of genetic services (7 items).

For each question on experience providing genetic services, outcome was expressed as a percentage (yes/no). Other answers were a mixture of 3–5-point Likert scales for attitude, knowledge, awareness and barriers. The 5-point Likert scale was collapsed for attitude section into negative (strongly disagree/disagree), moderate (neutral), and positive (agree/strongly agree) and responses reported in percentage. In the knowledge section, the 5-point-likert scale was collapsed into low knowledge (very low/low), average(average) and high knowledge (high, very high). The frequency at which barriers listed in the questionnaire were encountered by respondents was grouped into usually, sometimes, rarely and never.

## 3.8 Study Variables

- 1. **Rates of Genetic service provision-** Calculated by averaging responses to the 10 genetic service provision questions assessing family history taking, risk assessment, genetic counselling and genetic testing.
  2. Training in genetics- Defined as having had a course in medical genetics at school.
  3. Years in medical practice.
  4. Perceived availability of genetic services.
  5. Specialty
  6. Attitudes and perception towards genetics.
  7. Knowledge of Genetics.

## 3.9 Quality Assurance

Data was collected by the Principal Investigator. The survey employed a questionnaire for data collection that had been reviewed by a content specialist (medical geneticist) who verified its appropriateness for use. Permission for its use had been obtained from the authors and publishers. A detailed data collection plan that included specific data variables, sources and collection methods was developed. Potential biases or limitations in the data were identified and strategies to address them developed. A pilot study was conducted to pre-test, identify, and address any issues or challenges in data collection. This enabled testing of the data collection instruments procedures and protocols to refine them before actual data collection. Periodic checks were conducted in the google forms data sheet to ensure data quality and adherence to data collection guidelines.

## 3.10 Data Management and Analysis

A detailed documentation of the data collection process including protocols, forms is available in digital format and stored in a password protected computer accessible only to the Principal Investigator or to third parties with his authorization. Data was uploaded to Microsoft excel. Stored data was then exported to STATA version 14 for analysis.

Demographic characteristics were analysed and presented as frequencies and percentages for categorical data and as medians and range for continuous data. Proportion of physicians who provide basic genetic services will be presented as frequencies and percentage of total physicians surveyed. Attitudes and perceptions towards genetics and barriers to the provision of genetic services and knowledge of genetics was collected in a 5-point likert scale.

Each response was to be analysed individually as either positive or negative attitude and then scored as an aggregate score using the original Bloom’s cutoff. There was a total of 10 questions each with a score of 1-5, i.e., a minimum of 10 and maximum of 50. Using Bloom’s cut off (42), attitude was categorized as positive /good >80% (40-50), neutral 60%-79%(30-39.5), and negative/poor <60%(<30 points).Knowledge domain was analysed as proportion using original Bloom’s cut off(42). A score <60% (< 27) being poor, >60%-79%(27-35.5 points) being moderate, and >80%(>36 points) being good knowledge.

The outcome variable, utilization of genetic services was defined as whether one had ordered carrier testing on parents of a child who was confirmed to have a genetic disorder and/or ordered a genetic test for genetic disorder. Factors associated with physicians’ use of genetic services; training in genetics, specialty, and number of years in practice – analysed using bivariate and multivariate logistic regression. Associations will be reported by use of odds ratios and respective 95% confidence interval. P-values of <0.05 considered as significant.

## 3.11 Ethical considerations.

The Principal Investigator sought ethical approval from KNH-UON Ethics committee. Approval was also sought from the National Commission of Science Permission was sought from administrations of the selected institutions to ensure that there was compliance with laid down research procedures and access of individual information within the hospital.

An Consent Form in English language was provided. It provided a description of nature of the study, risks, potential benefits, their obligations, and the names with addresses of the investigators to be contacted. Informed consent forms were in duplicate, one copy for the participant and one copy for the investigator.

There were no risks to study respondents as data collection was performed by trained study personnel and did not involve any intervention. The extracted information was anonymized, encrypted, and stored in a password protected computer only accessible to the Principal Investigator.

Adherence to provisions of Kenya Data Protection Act: The professional body (KMPDC) and UoN informed the subjects that they will be sharing their data with third parties at the point of collection. The strongest basis for collecting and using data for research is consent. Physician and trainee physicians’ list are contained in a public record (KMPDC Website). The results of the research will be published in a non-identifiable form.

# CHAPTER FOUR.

# RESULTS

### 4.1 Characteristics of the study participants.

The study interviewed 191 participants, and their demographic characteristics is as shown in Table 1. Fifty seven percent (57%) of the respondents were male, with majority (63%) in clinical practice for 9years or less. Most (87%) lacked formal training in genetics while 32% expressed a special interest in genetics.

Table 2: Demographic characteristics of the participants.

| **Variable** | **N = 190**^1^ |
| --- | --- |
| **Sex** |  |
| Female | 109 (57%) |
| Male | 81 (43%) |
| **Years in clinical practice** |  |
| 0-9 | 120(63%) |
| 10-19 | 56 (29%) |
| 20-29 | 8 (4.2%) |
| 30-39 | 6 (3.2%) |
| **Practice setting (multiple choice)** |  |
| Private hospital | 51 (27%) |
| Public hospital | 161 (85%) |
| Solo practice | 9 (4.8%) |
| **Specialty** |  |
| Cardiology | 9 (4.7%) |
| Dermatology | 8 (4.2%) |
| Endocrinology | 9 (4.7%) |
| Gastroenterology. | 1 (0.5%) |
| Infectious disease | 2 (1.1%) |
| Internal medicine (no sub-specialization) | 79 (42%) |
| Nephrology | 6 (3.2%) |
| Pulmonology | 2 (1.1%) |
| Registrar in internal medicine | 73 (38%) |
| Rheumatology | 1 (0.5%) |
| **Has training in genetics** (apart from embryology) | 25 (13%) |
| **Has a special interest in genetics** | 60 (32%) |
| ^1^n (%) | |

## 4.2 Current Practice characteristics.

Eighty percent (80%) reported that evaluation and diagnosis of patients with genetic disorders was part of their practice. 63% of respondents refer patients for counselling/testing but only 37% know where to refer patients of genetic evaluation.

Up to 29% discuss genetic variation in drug response with their patients (pharmacogenomics). Genetic counselling while 25% reported knowing what genetic counselling entails.

Figure 2: Current practice characteristics

Twenty-four percent (24%) of respondents have referred a patient for genetic testing or evaluation, while 31.6% have ordered a genetic test as illustrated in figures 3 and 4 below. Seven percent (7%) reported that they have ordered carrier testing and 38% evaluate the usefulness of a genetic test.

Figure 3:Proportion of physicians who have referred a patient for genetic evaluation.

Figure 4: Ordered Carrier testing

Table 2: Family History taking

| **Variable** | **N = 190**^1^ |
| --- | --- |
| **proportion of patients you complete a family history** |  |
| 0 | 2 (1.1%) |
| 25% | 33 (17%) |
| 50% | 45 (24%) |
| 75% | 69 (36%) |
| 100% | 41 (22%) |
| **How often family history is updated** |  |
| At every visit | 20 (11%) |
| Every 2-4 years | 13 (6.8%) |
| Every 5-10 years | 17 (8.9%) |
| Never | 99 (52%) |
| Yearly or at periodic exam | 41 (22%) |
| **Completion of a standard form or checklist** | 74 (39%) |
| **A two or three generation family history** | 104 (55%) |
| **The family’s ethnic background** | 116 (61%) |
| **Medical Risk factors** | 177 (93%) |
| ^1^n (%) | |

Fifty-eight percent (58%) of respondents complete a family history in 75-100% of their patients with majority (52%) failing to update it completely. The family history contains two or three generations (55%), family’s ethnic background (61%) and medical risk factors (93%).

### 4.3 Attitudes towards Genomic Medicine

The respondent’s attitude towards genomic medicine was scored in a Likert scale of strongly disagree, disagree, neutral, agree and strongly agree. The responses were further categorized into three groups of negative attitudes (strongly disagree and disagree), neutral attitude (neutral), and positive attitude (strongly agree and agree).

The physicians surveyed in this study had an overall positive attitude towards genomics at 60% as illustrated below table 3 below.

Table 3: Overall Attitude towards Genomic medicine

| **Category and scores (Bloom’s cut off)** | **n** | **%** | Mean (SD) |
| --- | --- | --- | --- |
| **Attitude** |  |  | 2.4 (0.4) |
| Positive Attitude (80% +) | 113 | 60% |  |
| Neutral (60-79%) | 60 | 31% |  |
| Negative Attitude (< 60%) | 17 | 19% |  |

Majority (73%) agreed that there is need to intergrate genomic medicine into their practice and that there is need to keep abreast with advances in genomic medicine (76%).

Correspondingly 77% feel that there are benefits to warrant testing for inherited adult-onset diseases (77%).

However, only 37% felt that genomics is an exciting part of their practice. Furthermore, the majority agreed that genomic medicine will make an important contribution in the management of pre-natal, paediatric, and adult conditions.

Table 4: Physicians attitudes towards genomic medicine in categories

| **Variable** | **N = 190**^1^ |
| --- | --- |
| **I find genetics and genomics an exciting part of my practice.** |  |
| Moderate | 81(43%) |
| Negative | 38(20%) |
| Positive | 71(37%) |
| **There is need to incorporate genomic medicine into my practice.** |  |
| Moderate | 30(16%) |
| Negative | 22(12%) |
| Positive | 138(73%) |
| **I need to keep up to date with advances in genomic medicine.** |  |
| Moderate | 29 (15%) |
| Negative | 17 (8.9%) |
| Positive | 144(76%) |
| **There are sufficient benefits to warrant testing for inherited adult-onset diseases** |  |
| Moderate | 26 (14%) |
| Negative | 17 (8.9%) |
| Positive | 147(77%) |
| **Advances in genomic medicine will improve my patients’ outcome** |  |
| Moderate | 26 (14%) |
| Negative | 11 (5.8%) |
| Positive | 153(81%) |
| **It is important for me to learn about personalized patient care based on targeted or whole genome sequencing** |  |
| Moderate | 33 (17%) |
| Negative | 17 (8.9%) |
| Positive | 140(74%) |
| **Genomic medicine is going to make important contributions in diagnosis and management of:**  **-Prenatal conditions** |  |
| Moderate | 13 (6.8%) |
| Negative | 12 (6.3%) |
| Positive | 165(87%) |
| **-Pediatric conditions** |  |
| Moderate | 18 (9.5%) |
| Negative | 11 (5.8%) |
| Positive | 161(85%) |
| **-Adult-onset conditions.** |  |
| Moderate | 20 (11%) |
| Negative | 16 (8.4%) |
| Positive | 154(81%) |
| **Parents should be able to have their children (under 18) tested for susceptibility to adult-onset genetic diseases.** |  |
| Moderate | 45 (24%) |
| Negative | 22 (12%) |
| Positive | 123(65%) |
| **I can identify useful sources of information regarding genetics in my practice** |  |
| Moderate | 62 (33%) |
| Negative | 61 (32%) |
| Positive | 67 (35%) |
| **I can find information about the genetic tests available within our health system** |  |
| Moderate | 46 (24%) |
| Negative | 115(61%) |
| Positive | 29 (15%) |
| **I know how to contact my local genetics center.** |  |
| Moderate | 32 (17%) |
| Negative | 145(76%) |
| Positive | 13 (6.8%) |
| ^1^n (%) | |

### 4.4 Knowledge of genetic disorders and awareness of genetic services.

#### 4.4.1 Knowledge of Genetics

Respondents were asked to grade their knowledge in various Knowledge outcome aggregate scores were categorized using Bloom’s cut off (<60% was low, 60-79% moderate/average, >80% high. Overall, majority of respondents (64%) graded their knowledge of various aspects of genetics as moderate with only 7% having high level knowledge as illustrated below.

Table 5: Overall knowledge of genetics

| **Category and scores (Bloom’s cut off)** | **n** | **%** | Mean (SD) |
| --- | --- | --- | --- |
| **Knowledge** |  |  | 1.7 (0.45) |
| High level (80% +) | 13 | 7% |  |
| Moderate level (60-79%) | 121 | 64% |  |
| Low level (< 60%) | 56 | 29% |  |

Knowledge of emerging areas of genomic medicine such as newer technologies entering clinical practice (low-68%), interpreting results of ‘direct to consumer’ tests. (low-71%) was noticeably low.

Table 6:Physicians' Self-perceived knowledge of genetics in categories

| **Variable** | **N = 190**^1^ |
| --- | --- |
| **Genetic consequences of consanguinity** |  |
| Moderate knowledge | 99(52%) |
| **Ethnic-specific disorders (e.g., hemoglobinopathies, Tay-Sachs disease, cystic fibrosis)** |  |
| Moderate knowledge | 107(56%) |
| **Genetic testing of minors for adult-onset disorders** |  |
| Low knowledge | 105(55%) |
| **Genomic risk factors for common complex disorders (e.g., cancer, heart diseases, diabetes)** |  |
| Moderate knowledge | 102(54%) |
| **Genetics of common single gene disorders (e.g. cystic fibrosis, hereditary breast and ovarian cancer)** |  |
| Moderate knowledge | 96(51%) |
| **New genomic technologies entering clinical practice e.g whole genome sequencing, microarray, direct to consumer genomic testing** |  |
| Low knowledge | 130(68%) |
| **Interpreting results of ‘direct to consumer’ genomic tests.** |  |
| Low knowledge | 134(71%) |
| **Basic genetic concepts (e.g. inheritance, genes, mutation, penetrance, predisposition vs diagnosis)** |  |
| Moderate knowledge | 76(40%) |
| **Genetic variation in drug response e.g pharmacogenetics.** |  |
| Low knowledge | 95(50%) |
| ^1^n (%) | |

#### 4.4.2. Awareness of genetic services.

There were 3 items evaluating physicians’ awareness of local genetic services. This was collected in a 5-point Likert scale and collapsed in low awareness (strongly disagree/disagree), moderate awareness (neutral) and aware (agree/strongly agree). Majority (76%) do not know how to contact their contact their local genetics facility or obtain information about genetic tests within the health system. (61%).

Table 7: Awareness of Genetic services.

| **Identification of useful sources of information regarding genetics in clinical practice** |
| --- |
| Moderate 62(33%) |
| Negative 61(32%). |
| Positive 67(35%) |
| **I can find information about the genetic tests available within our health system** |
| Moderate 46(24%) |
| Negative 115(61%) |
| Positive 29(15%) |
| **I know how to contact my local genetics center.** |
| Moderate 32(17%) |
| Negative 145(76%) |
| Positive 13(6.8%) |

### 4.5. Barriers to the provision of genetic services

In this study physicians identified limited access to medical geneticist (80%), absence of referral guidelines (86%), high cost of genomic services (93%), inadequate knowledge of genomic medicine and limited access to genetic testing services as barriers to the provision of genetic services.

Table 8: Barriers to the provision of genetic services

| **Variable** | **N = 190**^1^ |
| --- | --- |
| **Limited access to a medical geneticist for referrals for testing and/or consultation** |  |
| Usually | 152 (80%) |
| **Lack of referral guidelines** |  |
| Usually | 163 (86%) |
| **High cost of genetic services such as testing.** |  |
| Usually | 177 (93%) |
| **Inadequate knowledge of genetics, genetic testing and genetic counselling.** |  |
| Usually | 117 (62%) |
| **Lack of patient interest in genetic evaluation.** |  |
| Usually | 78 (41%) |
| **Limited access to genetic testing services** |  |
| Usually | 155 (82%) |
| **Lack of detailed or updated family history** |  |
| Usually | 88 (46%) |
| ^1^n (%) | |

### 4.6 Correlation between physician characteristics and their utilization of genetic services.

Regression analysis was done for the secondary objective. The outcome variables analysed were whether one had ever:

-Ordered carrier testing on parents of a child who was confirmed to have a genetic disorder.

-Ordered a genetic test for genetic disorder.

There was a weak positive correlation between the years in clinical practice and the outcome variables above (p-value 0.018). Respondents who had training in genetics were also more likely to have ordered carrier testing on parents of a child who was confirmed to have a genetic disorder or ordered genetic test for a genetic disorder (p-value 0.037).

Years in clinical practice and whether one had received training were fit in the multivariate model. It was found that doctors who had more than 9 years of clinical practice were more likely to utilize genetic services compared to those with less than 9 years (adjusted OR = 1.16, 95% CI [1.16 – 4.09], p = 0.016]. Doctors who received training in genetics were more than twice likely to utilize genetic services compared to those who did not (aOR = 2.60, 95% CI [1.09- 6.29], p = 0.031).

Table 9: Correlation between selected physician characteristics and outcome variables.

|  | **Univariate** | | | | | **Multivariate** | | |
| --- | --- | --- | --- | --- | --- | --- | --- | --- |
| **Characteristic** | **N** | **Event N** | **OR**^1^ | **95% CI**^1^ | **p-value** | **OR**^1^ | **95% CI**^1^ | **p-value** |
| **Sex** | 190 | 63 |  |  | 0.51 |  |  |  |
| Female |  |  | *Ref.* | — |  |  |  |  |
| Males |  |  | 1.23 | 0.67- 2.26 |  |  |  |  |
| **Years in clinical practice** | 190 | 63 |  |  | **0.018** |  |  |  |
| <9 |  |  | *Ref.* | — |  | *Ref.* | — |  |
| 9 + |  |  | 2.11 | 1.13- 3.93 |  | 2.17 | 1.16-4.09 | **0.016** |
| **Practice setting** | 190 | 63 |  |  | 0.60 |  |  |  |
| Solo practice |  |  | *Ref.* | — |  |  |  |  |
| Public and Private hospitals |  |  | 1.57 | 0.54- 4.72 |  |  |  |  |
| Public hospital |  |  | 1.04 | 0.45-2.57 |  |  |  |  |
| **Specialty** | 190 | 63 |  |  | 0.43 |  |  |  |
| Internal Medicine registrar |  |  | *Ref.* | — |  |  |  |  |
| Internal Medicine specialist |  |  | 1.07 | 0.54- 2.15 |  |  |  |  |
| Sub-specialist |  |  | 1.69 | 0.74-3.82 |  |  |  |  |
| **Have any training in genetics? (apart from embryology)** | 190 | 63 |  |  | **0.037** |  |  |  |
| No |  |  | *Ref.* | — |  | *Ref.* | — |  |
| Yes |  |  | 2.49 | 1.06- 5.92 |  | 2.60 | 1.09-6.29 | **0.031** |
| **Have a special interest in genetics** | 190 | 63 |  |  | 0.77 |  |  |  |
| No |  |  | *Ref.* | — |  |  |  |  |
| Yes |  |  | 0.91 | 0.46-1.73 |  |  |  |  |
| ^1^OR = Odds Ratio, CI = Confidence Interval | | | | | | | | |

# CHAPTER FIVE

**DISCUSSION, CONCLUSION AND RECOMMENDATIONS**

## 5.1 Discussion

Genomic medicine is expected to greatly affect clinical practice in the near future. Kenya is undergoing an epidemiological transition with the increasing prevalence of non-communicable diseases expected to the expand the role of genomic medicine. The aim of this study was to offer the first comprehensive view of physicians’ involvement in genomic medicine, their awareness of currently available genetic services, attitudes towards genomic medicine and barriers to the integration of genomic medicine into mainstream healthcare in Kenya.

Some key and core competencies in genomic medicine that can be undertaken by physicians have been defined (43). In Kenya and Africa at large there has been limited research to evaluate delivery of genetic services at clinical level. In this study majority of the participating physicians reported that they undertake clinical aspects of genomic medicine such as evaluation and diagnosis of patients with genetic disorders, referral of patients for specialised genetic services, and providing education about genetic conditions. This indicates that basic genetic principles are incorporated in medical practice. Other studies have reported similar results; from a Canadian survey of family physicians (44), Suchard et al(45);and Hayflick et al(18) .

Involvement in areas such as genetic counselling, pharmacogenomics is however low. It is debatable whether physicians should provide counselling to patients and families susceptible to inherited diseases. In the absence of professional genetic counsellors, the role of physicians in counselling is integral. These findings are similar to those in literature. Haga et al(46) in a survey of primary care physicians found that only 13 % of respondents felt comfortable ordering pharmacogenomic tests while a quarter reported not having any education about pharmacogenetics. Capacity building and training is needed in these areas through continuous medical education as they are not incorporated into traditional medical education.

Worryingly, 68% of physicians reported to have never ordered a genetic test for a genetic disorder. In addition, majority reported that they do not discuss benefits, risks, or limitations of genetic tests with patients or provide support to patients coping with genetic results. Even fewer knew where to refer patients for genetic evaluation. The low uptake of genetic testing in our setting could be due to lack of training relevant to genetic testing, as well lack of knowledge and experience and limited testing infrastructure. The literature is mixed in this regard. In a 2008 survey of Primary care physicians in the US 60% of physicians had ordered a genetic test (28). Genetic testing has the potential to decrease morbidity and mortality of chronic diseases especially when used for risk profiling early in the management (28).

Family history is a basic tool in clinical genetics and is essential in determining risk and identifying individuals who might benefit from additional genetic evaluation (22). Key in family history taking is collecting adequate amounts of information and proper interpretation. Fifty-eight 58% of physicians reported that they collect family history in 75%-100% of patients seen, with majority incorporating two or three generations (55%), family’s ethnic background (61%) and medical risk factors (93%). Previous surveys among physicians have shown an imbalance between frequency and quality of family history taking(44)(47). Development of standardized family history taking tools with checklists and emphasis on proper history taking as part of medical education can be used to improve existing knowledge and proficiency in eliciting family history.

The study also looked at physicians’ attitude towards genomic medicine. About 60% of physicians surveyed in this study had a positive attitude towards genomic medicine. More than two-thirds of respondents felt that there is need to integrate genetics into their practice and keep up to date with advances in genomic medicine. Over half of the respondents were certain that advancements in genomic medicine would enhance patient outcomes and that there are sufficient benefits to warrant testing for inherited adult-onset diseases. However, majority do not find genetics and genomics an important part of their practice. This can be leveraged to enhance integration of genomic medicine into mainstream healthcare. In a South African study examining knowledge and attitudes towards predictive testing, attitude was largely positive despite the costs as they perceived benefits outweighed costs (48). These findings also supports existing literature where participants felt that genomic medicine is likely to influence clinical practice in future (44) (29)(48).

Knowledge of genetics has been found to translate into increased uptake of genomic medicine into mainstream healthcare(48). Most (64%) of respondents rated their overall knowledge in genomic medicine as moderate i.e. 60-79% on bloom’s cutoff. Knowledge in basic concepts such as genetic consequences of consanguinity and genetic risk factors common complex disorders as moderate to high. Knowledge of advanced/emerging themes such as interpretation of direct-to-consumer testing and pharmacogenomics was largely poor. Specific educational interventions should be geared towards these areas. Self-perceived knowledge deficits are a global problem(49)(50). More data is needed to determine specific educational resource needs and develop genetic educational programmes targeting physicians.

Systemic and individual barriers to providing genetic services have been previously identified in literature (38)(6)(51). In this study physicians identified limited access to medical geneticist (80%), lack of referral guidelines (86%), high cost of genetic services (93%), inadequate knowledge of genetics among others. These barriers impact practice, further impeding care delivery to patients in need of genetic services. Based on these findings multiple strategies can be deployed to ameliorate access to genetic services as enumerated below.

This needs assessment survey provides important insights into the state of genomic medicine in Kenya. The lack of awareness of existing genetic services and low uptake of genetic testing and counselling shows the need for investment in infrastructure such as genomic labs and training of personnel such as medical geneticists and genetic counsellors. There is need to develop genetics-training programmes and further incorporate genetics into the medical curriculum to improve the knowledge, skills, and confidence of physicians in genomic medicine.

## 5.2 Conclusion

Evaluation and diagnosis of patients with genetic conditions and referral of patients for genetic evaluation were part of majority of respondents’ practice. However, there is low uptake of genetic testing, genetic counselling and pharmacogenomics. The physicians had a positive attitude towards genomic medicine. Barriers to access/provision of genetic services were limited access to medical geneticists for referral, high cost of genetic services such as testing, inadequate knowledge of genetics and limited access to genetic services. Despite progress, challenges remain in integration of genomic medicine into clinical practice.

## 5.3 Recommendations

Study provides important insights into the state of genomic medicine in Kenya. Based on our findings, we recommend the following to improve the utilisation of genomic services among physicians in Kenya

1. Development of genetics training programmes and further incorporation of genetics into the medical curriculum.
2. Investment in infrastructure such as genomic testing laboratories and training of personnel such as medical geneticists and genetic counsellors/

Future research can be conducted among other stakeholders (patients, other medical disciplines) to provide more conclusive inference

## 5.4 Study strengths

This study used the KMPDC database and list of registrars obtained from the university of Nairobi which captures physicians practicing in Kenya.

This is the first study done to determine physician’s uptake and utilisation of genomic services.

## 5.5 Study Limitations

Possible response bias due to self-reporting. This was addressed by wording questions neutrally and ensuring answer options were open-ended.

Despite attaining the desired sample size of 191, overall response rate was low especially among practicing physicians (117/394) bringing into question the generalizability of the results.

In addition, trainee physicians from other universities could not be surveyed as necessary approvals could not be obtained on time.

We relied on physicians’ self-report to assess their experience practicing genomic medicine. This may differ from actual physician practice.

## 5.6 Study Significance and Results Dissemination

We were able to identify gaps in physician knowledge regarding basic genetic concepts and their utilisation and provision of genetic services. These data will have implications for medical practice, research, policy, and education in this rapidly expanding area.

Future research can be conducted among other stakeholders (patients, other medical disciplines) to provide more conclusive inference.

# REFERENCES

1. Manolio TA, Chisholm RL, Ozenberger B, Roden DM, Williams MS, Wilson R, et al. Implementing genomic medicine in the clinic: the future is here. Genet Med. 2013 Apr;15(4):258–67.

2. Khoury MJ. Genetics and genomics in practice: The continuum from genetic disease to genetic information in health and disease. Genet Med. 2003 Jul 1;5(4):261–8.

3. Verma IC, Puri RD. Global burden of genetic disease and the role of genetic screening. Semin Fetal Neonatal Med. 2015 Oct;20(5):354–63.

4. Ballantyne A, Goold I, Pearn A, Programme WHG. Medical genetic services in developing countries : the ethical, legal and social implications of genetic testing and screening [Internet]. World Health Organization; 2006 [cited 2023 May 25]. Available from: https://apps.who.int/iris/handle/10665/43288

5. Hawkins AK, Hayden MR. A grand challenge: Providing benefits of clinical genetics to those in need. Genet Med. 2011 Mar 1;13(3):197–200.

6. Suther S, Goodson P. Barriers to the provision of genetic services by primary care physicians: A systematic review of the literature. Genet Med. 2003 Mar 1;5(2):70–6.

7. Dragojlovic N, Kopac N, Borle K, Tandun R, Salmasi S, Ellis U, et al. Utilization and uptake of clinical genetics services in high-income countries: A scoping review. Health Policy. 2021 Jul 1;125(7):877–87.

8. Truong TK, Kenneson A, Rosen AR, Singh RH. Genetic Referral Patterns and Responses to Clinical Scenarios: A Survey of Primary Care Providers and Clinical Geneticists. J Prim Care Community Health. 2021 Sep 29;12:21501327211046734.

9. Chong JX, Buckingham KJ, Jhangiani SN, Boehm C, Sobreira N, Smith JD, et al. The Genetic Basis of Mendelian Phenotypes: Discoveries, Challenges, and Opportunities. Am J Hum Genet. 2015 Aug 6;97(2):199–215.

10. Wanjiku CM, Njuguna F, Chite Asirwa F, Mbunya S, Githinji C, Roberson C, et al. Establishing care for sickle cell disease in western Kenya: achievements and challenges. Blood Adv. 2019 Dec 6;3(Suppl 1):8–10.

11. Burton H, Cole T, Lucassen A. Genomic medicine: challenges and opportunities for physicians. Clin Med. 2012 Oct;12(5):416–9.

12. Watson JD, Crick FHC. The Structure of Dna. Cold Spring Harb Symp Quant Biol. 1953 Jan 1;18:123–31.

13. Verma M, Kulshrestha S, Puri A. Genome Sequencing. In: Keith JM, editor. Bioinformatics: Volume I: Data, Sequence Analysis, and Evolution [Internet]. New York, NY: Springer; 2017 [cited 2023 Jul 10]. p. 3–33. (Methods in Molecular Biology). Available from: https://doi.org/10.1007/978-1-4939-6622-6_1

14. Lander ES, Linton LM, Birren B, Nusbaum C, Zody MC, Baldwin J, et al. Initial sequencing and analysis of the human genome. Nature. 2001 Feb;409(6822):860–921.

15. Naidoo N, Pawitan Y, Soong R, Cooper DN, Ku CS. Human genetics and genomics a decade after the release of the draft sequence of the human genome. Hum Genomics. 2011 Oct 1;5(6):577–622.

16. GOV.UK [Internet]. [cited 2023 May 26]. Genome UK: shared commitments for UK-wide implementation 2022 to 2025. Available from: https://www.gov.uk/government/publications/genome-uk-shared-commitments-for-uk-wide-implementation-2022-to-2025/genome-uk-shared-commitments-for-uk-wide-implementation-2022-to-2025

17. Hull LE, Gold NB, Armstrong KA. Revisiting the Roles of Primary Care Clinicians in Genetic Medicine. JAMA. 2020 Oct 27;324(16):1607–8.

18. Hayflick SJ, Patrice Eiff M, Carpenter L, Steinberger J. Primary care physicians’ utilization and perceptions of genetics services. Genet Med. 1998 Nov;1(1):13–21.

19. Genetic Testing | NEJM [Internet]. [cited 2023 May 26]. Available from: https://www.nejm.org/doi/full/10.1056/nejmoa012113

20. A New Definition of Genetic Counseling: National Society of Genetic Counselors’ Task Force Report - Resta - 2006 - Journal of Genetic Counseling - Wiley Online Library [Internet]. [cited 2023 May 26]. Available from: https://onlinelibrary.wiley.com/doi/10.1007/s10897-005-9014-3

21. Nelson HD, Pappas M, Cantor A, Haney E, Holmes R. Risk Assessment, Genetic Counseling, and Genetic Testing for BRCA-Related Cancer in Women: Updated Evidence Report and Systematic Review for the US Preventive Services Task Force. JAMA. 2019 Aug 20;322(7):666–85.

22. Ginsburg GS, Wu RR, Orlando LA. Family health history: underused for actionable risk assessment. The Lancet. 2019 Aug 17;394(10198):596–603.

23. Alliance G, ScreeningServices TNYMAC for G and N. PEDIGREE AND FAMILY HISTORY-TAKING. In: Understanding Genetics: A New York, Mid-Atlantic Guide for Patients and Health Professionals [Internet]. Genetic Alliance; 2009 [cited 2023 Sep 18]. Available from: https://www.ncbi.nlm.nih.gov/books/NBK115557/

24. Health I of M (US) R on TGBR for. Genetic Service Delivery: The Current System and Its Strengths and Challenges. In: Innovations in Service Delivery in the Age of Genomics: Workshop Summary [Internet]. National Academies Press (US); 2009 [cited 2023 May 26]. Available from: https://www.ncbi.nlm.nih.gov/books/NBK26394/

25. He LQ, Njambi L, Nyamori JM, Nyenze EM, Kimani K, Matende I, et al. Developing Clinical Cancer Genetics Services in Resource-Limited Countries: The Case of Retinoblastoma in Kenya. Public Health Genomics. 2014;17(4):221–7.

26. Hofman KJ, Tambor ES, Chase GA, Geller G, Faden RR, Holtzman NA. Physicians’ knowledge of genetics and genetic tests. Acad Med. 1993 Aug;68(8):625.

27. Lee S, Gedleh A, Hill JA, Qaiser S, Umukunda Y, Odiyo P, et al. In Their Own Words: A Qualitative Study of Kenyan Breast Cancer Survivors’ Knowledge, Experiences, and Attitudes Regarding Breast Cancer Genetics. J Glob Oncol. 2017 Dec 4;4:JGO.17.00061.

28. Alevandra E. Shields, PHD, Wylie Burke ,MD, PHD, and Douglas E. Levy, PHD. Differential Use of Available Genetic Tests among Primary Care Physiscians in the U.S. Genet Med.

29. Klitzman R, Chung W, Marder K, Shanmugham A, Chin LJ, Stark M, et al. Attitudes and Practices Among Internists Concerning Genetic Testing. J Genet Couns. 2013;22(1):90–100.

30. Thom J, Haw T. Awareness of genetic counseling services among allied healthcare professionals in South Africa. J Genet Couns. 2021;30(6):1649–57.

31. Tung N. Management of Women With BRCA Mutations : A 41-Year-Old Woman With a BRCA Mutation and a Recent History of Breast Cancer. JAMA. 2011 Jun 1;305(21):2211–20.

32. Pitceathly RDS. Mitochondrial Extrapyramidal Syndromes: Using Age and Phenomenology to Guide Genetic Testing. JAMA Neurol. 2016 Jun 1;73(6):630–2.

33. Wolf MJ, Noeth D, Rammohan C, Shah SH. The Complexities of Genetic Testing in Familial Dilated Cardiomyopathy. Circ Cardiovasc Genet. 2016 Feb;9(1):95–9.

34. Bellcross CA, Kolor K, Goddard KAB, Coates RJ, Reyes M, Khoury MJ. Awareness and Utilization of BRCA1/2 Testing Among U.S. Primary Care Physicians. Am J Prev Med. 2011 Jan 1;40(1):61–6.

35. Menasha JD, Schechter C, Willner J. Genetic testing: a physician’s perspective. Mt Sinai J Med N Y. 2000 Mar;67(2):144–51.

36. Christensen KD, Vassy JL, Jamal L, Lehmann LS, Slashinski MJ, Perry DL, et al. Are Physicians Prepared for Whole Genome Sequencing? A Qualitative Analysis. Clin Genet. 2016 Feb;89(2):228–34.

37. Rich EC, Burke W, Heaton CJ, Haga S, Pinsky L, Short MP, et al. Reconsidering the Family History in Primary Care. J Gen Intern Med. 2004 Mar;19(3):273–80.

38. Zhong A, Xia K, Hadjis Z, Lifman G, Njambi L, Dimaras H. Opportunities and barriers for genetic service delivery in Kenya from a health personnel perspective. J Community Genet. 2021 Oct;12(4):525–38.

39. Introducing medical genetics services in Ethiopia using the MiGene Family History App - Genetics in Medicine [Internet]. [cited 2023 Sep 12]. Available from: https://www.gimjournal.org/article/S1098-3600(21)04634-7/fulltext

40. Baldwin LM, Trivers KF, Andrilla CHA, Matthews B, Miller JW, Lishner DM, et al. Accuracy of Ovarian and Colon Cancer Risk Assessments by U.S. Physicians. J Gen Intern Med. 2014 May 1;29(5):741–9.

41. KMPDC [Internet]. [cited 2023 Jun 18]. Available from: http://kmpdc.go.ke/Registers/Specialist_Practitioners.php

42. Alzahrani MM, Alghamdi AA, Alghamdi SA, Alotaibi RK. Knowledge and Attitude of Dentists Towards Obstructive Sleep Apnea. Int Dent J. 2021 Jun 28;72(3):315–21.

43. Korf BR, Berry AB, Limson M, Marian AJ, Murray MF, O’Rourke PP, et al. Framework for development of physician competencies in genomic medicine: report of the Competencies Working Group of the Inter-Society Coordinating Committee for Physician Education in Genomics. Genet Med. 2014 Nov 1;16(11):804–9.

44. Carroll JC, Allanson J, Morrison S, Miller FA, Wilson BJ, Permaul JA, et al. Informing Integration of Genomic Medicine Into Primary Care: An Assessment of Current Practice, Attitudes, and Desired Resources. Front Genet. 2019 Nov 21;10:1189.

45. Suchard MA, Yudkin P, Sinsheimer JS, Fowler GH. General practitioners’ views on genetic screening for common diseases. Br J Gen Pract. 1999 Jan;49(438):45–6.

46. Haga SB, Burke W, Ginsburg GS, Mills R, Agans R. Primary Care Physicians’ Knowledge of and Experience with Pharmacogenetic Testing. Clin Genet. 2012 Oct;82(4):388.

47. Sussner KM, Jandorf L, Valdimarsdottir HB. Educational needs about cancer family history and genetic counseling for cancer risk among frontline healthcare clinicians in New York City. Genet Med. 2011 Sep 1;13(9):785–93.

48. Naidoo L, Reddy P. Stakeholder knowledge and attitudes toward the use of predictive genetic testing in South Africa. J Community Genet. 2022 Dec;13(6):567–77.

49. Li J, Xu T, Yashar BM. Genetics educational needs in China: physicians’ experience and knowledge of genetic testing. Genet Med. 2015 Sep;17(9):757–60.

50. Use of Genetic Tests among Neurologists and Psychiatrists: Knowledge, Attitudes, Behaviors, and Needs for Training | Journal of Genetic Counseling [Internet]. [cited 2024 May 7]. Available from: https://link.springer.com/article/10.1007/s10897-013-9624-0

51. Najafzadeh M, Davis JC, Joshi P, Marra C. Barriers for integrating personalized medicine into clinical practice: a qualitative analysis. Am J Med Genet A. 2013 Apr;161A(4):758–63.

##

# APPENDICES

## Appendix A: Participants Consent

**Research Title:** Utilisation of genomic services among Kenyan Physicians.

**Primary Investigator:** Dr Sigilai K.Hillary (Reg no: H58/40563/2021)

Master of Medicine in Internal Medicine

University of Nairobi

**Supervisors:** Prof. Elijah N Ogola

-Consultant physician and cardiologist

Dr Syokau Ilovi

-Consultant physician and geneticist

**Participants:** Physicians practicing across Kenya.

**What is the purpose of this survey?**

The delivery of genetic services is crucial in the management of heritable disorders, diseases, and syndromes. In Kenya provision of clinical genetic services remains limited but their availability has started to increase. This study will examine their uptake, quality, and accessibility from the viewpoint of medical practitioners, specifically physicians. We, therefore, like to invite you to participate in the research. You should only participate if you want to and, it is important to read the following information carefully and ask if there is anything that is not clear or if you would like more information.

**Broad Objective:** To describe the delivery of basic genetic services among physicians and factors affecting integration of genomic medicine into their practice.

**Benefits of the study.**

The resultant findings will identify gaps in the provision of clinical genetic services, and thus provide a basis for future research, policy, and education in this rapidly expanding area.

**Procedures**

If you choose to participate in this study, a unique random number shall be assigned to you to hide your identity throughout the study. The response to your questionnaire form shall only be accessed by authorized personnel and it will only be used for statistical data analysis.

**Confidentiality**

Informed consent will be obtained from the participants prior to conducting the study. Confidentiality will be maintained during the study. Only the Principal Investigator will have access to the data for the duration of the study. At the end of the project, all the data will be submitted to University of Nairobi Faculty of Health Sciences as per institutional policies.

**Risk of participation:** There are no specific dangers, risks or adverse consequences associated with your decision to participate in this study.

**Participant Rights:** Your rights as a participant are.

- Your participation in this study is entirely voluntary.

- You can also choose to withdraw from the study at any time without necessarily giving a reason for your withdrawal.

-You will not receive any payment to participate in this study.

**Participant’s statement**

I, the participant, have been explained to and have understood, and willingly accept to participate in the study.

Participant signature / Thumb stamp _______________________ Date _______________

**Researcher’s statement.**

I, the researcher having explained the motive of this study, hereby commit that secrecyof the information collected will be maintained.

Researcher ‘s Name: _____________________________________ Date: _______________

Signature____________________________________________________________________

Role in the study: ___________________________ [i.e., study staff who explained the informed consent form.]

Principal Investigator: Dr Sigilai.K.Hillary

Email: shsigihillary@gmail.com

Tel: 0719512129

Supervisors

Prof. Elijah N. Ogola

Consultant Cardiologist

Email: elijah.ogola@uonbi.ac.ke

Tel: 0722 737944

Dr. Syokau Ilovi

Consultant Physician and Geneticist

Email: [csilovi@uonbi.ac.ke](mailto:csilovi@uonbi.ac.ke)

Tel: 0722 233157

KNH-UoN ERC

Email: [uonknh_erc@uonbi.ac.ke](mailto:uonknh_erc@uonbi.ac.ke)

Website: <http://www.erc.uonbi.ac.ke>

##

## Appendix B: Questionnaire

**Section A: Genetic service in current primary care practice.**

**Please indicate which tasks are part of your current practice.**

|  | **Task** | **Is this part of your current practice?** | |
| --- | --- | --- | --- |
|  |  | **Yes** | **No** |
| QN |  |  |  |
| i | Eliciting information about genetic conditions as part of family or medical history. |  |  |
| ii | Identifying individuals with genetic disorders. |  |  |
| iii | Deciding who should be offered referral for genetic counselling or testing based on personal or family health history. |  |  |
| iv | Knowing where to refer for genetic counselling/testing |  |  |
| v | Providing support to patients coping with genetic results. |  |  |
| vi | Evaluating clinical usefulness of a genetic test. |  |  |
| vii | Discussing benefits, risks, limitations of a genetic test with patients. |  |  |
| viii | Describing what to expect at a genetic counselling session |  |  |
| ix | Obtaining credible, current information about genetics. |  |  |
| x. | Providing education about genetic condition to patients. |  |  |
|  |  |  |  |

**Section B: Family History**

B1. For what proportion of patients do you complete a family history/

1. 0% b. 25%. c. 50%. d. 75% e. 100%

B2. How often do you routinely update family history? (excluding new personal or family health issues that come up at a visit) (***circle only one)***

i. Never. ii. Every 5-10 years iii. Every 2-4 years. iv. Yearly or at periodic exam. v. At every visit

B3. What does the family history usually include?

YES NO

1. Completion of a standard form or checklist 1 2
2. A two or three generation family history 1 2
3. The family’s ethnic background. 1 2
4. Medical Risk factors. 1 2

**Section C: Attitude towards genomic medicine.**

The term genome refers to an individual’s entire unique DNA sequence. **Genomics** is the study of genes their function and interaction with all other genes in the genome and with their environment. **Genomic medicine** uses an individual’s risk, predisposition, diagnosis and prognosis, and the selection and prioritization of therapeutic options. (e.g., pharmacogenetic testing prior to administration of certain medications.)

| QN |  | **Strongly disagree** |  | **Neutral** |  | **Strongly agree** |
| --- | --- | --- | --- | --- | --- | --- |
| i | I find genetics and genomics an exciting part of my practice. | 1 | 2 | 3 | 4 | 5 |
| ii | There is need to incorporate genomic medicine into my practice. | 1 | 2 | 3 | 4 | 5 |
| iii | I need to keep up to date with advances in genomic medicine. | 1 | 2 | 3 | 4 | 5 |
| iv | There are sufficient benefits to warrant testing for inherited adult-onset diseases. | 1 | 2 | 3 | 4 | 5 |
| v | Advances in genomic medicine will improve my patients’ outcome. | 1 | 2 | 3 | 4 | 5 |
| vi | It is important for me to learn about personalized patient care based on targeted or whole genome sequencing. | 1 | 2 | 3 | 4 | 5 |
| vii | Genomic medicine is going to make important contributions in diagnosis and management of:  -Prenatal conditions  -pediatric conditions  -Adult-onset conditions. | 1  1  1 | 2  2  2 | 3  3  3 | 4  4  4 | 5  5  5 |
| vii | Parents should be able to have their children (under 18) tested for susceptibility to adult-onset genetic diseases. | 1 | 2 | 3 | 4 | 5 |
|  |  |  |  |  |  |  |

**SECTION D: Awareness of genetic services**

|  |  |  |  |  |  |  |
| --- | --- | --- | --- | --- | --- | --- |
| QN |  | Strongly  Disagree |  | Neutral |  | Strongly  Agree |
| D1 | I can identify useful sources of information regarding genetics in my practice | 1 | 2 | 3 | 4 | 5 |
| D2 | I can find information about the genetic tests available within our health system | 1 | 2 | 3 | 4 | 5 |
| D3 | I know how to contact my local genetics center. | 1 | 2 | 3 | 4 | 5 |
|  |  |  |  |  |  |  |

**SECTION E: Experience With Genetic Services.**

E1. Do you know where to refer patients for genetic testing/evaluation?

a.Yes. b. No

E2. Have you ever referred a patient for genetic testing/evaluation?

a.Yes. b. No

E3. How confident do you feel dealing with genetic disorders?

a.Very confident. b. Neutral. c. Not at all confident.

E4. How would you rate your knowledge of genetics and genetic disorders?

a.Very high. b. Average. c. Very Low

E5. Have you ever ordered carrier testing on parents of a child who was confirmed to have cystic fibrosis or a haemoglobinopathy on newborn screening?

a.Yes. if yes how many times this year?

b. No

E6. Have you ever ordered a genetic test for a genetic disorder?

a.Yes, if yes how many?

b. No

**SECTION F.**

How would you rate your knowledge of?

|  |  |  |  |  |  |  |
| --- | --- | --- | --- | --- | --- | --- |
| QN |  | Very low |  | Average |  | Very High |
| F1 | Genetic consequences of consanguinity | 1 | 2 | 3 | 4 | 5 |
| F2 | Ethnic-specific disorders (e.g., hemoglobinopathies, Tay-Sachs disease, cystic fibrosis) | 1 | 2 | 3 | 4 | 5 |
| F3 | Genetic testing of minors for adult-onset disorders | 1 | 2 | 3 | 4 | 5 |
| F4 | Genomic risk factors for common complex disorders (e.g., cancer, heart diseases, diabetes) | 1 | 2 | 3 | 4 | 5 |
| F5 | Genetics of common single gene disorders (e.g. cystic fibrosis, hereditary breast and ovarian cancer) | 1 | 2 | 3 | 4 | 5 |
| F6 | New genomic technologies entering clinical practice e.g whole genome sequencing, microarray, direct to consumer genomic testing | 1 | 2 | 3 | 4 | 5 |
| F7 | Interpreting results of ‘direct to consumer’ genomic tests. | 1 | 2 | 3 | 4 | 5 |
| F8 | Basic genetic concepts (e.g. inheritance, genes, mutation, penetrance, predisposition vs diagnosis) | 1 | 2 | 3 | 4 | 5 |
| F9 | Genetic variation in drug response e.g pharmacogenetics. | 1 | 2 | 3 | 4 | 5 |
|  |  |  |  |  |  |  |

**SECTION G. Barriers to delivery of genetic services**

| QN |  | Usually | Sometimes | Rarely | Never |
| --- | --- | --- | --- | --- | --- |
| i | Limited access to geneticist for referrals for testing and/or consultation. |  |  |  |  |
| ii | I don’t see much benefit for the patient |  |  |  |  |
| iii | Lack of referral Guidelines |  |  |  |  |
| iv | High cost of services |  |  |  |  |
| v | Lack of training/ practice guidelines on genetic testing interpretation |  |  |  |  |
| vi | Lack of patient interest |  |  |  |  |
| vii | Limited access to genetic testing services. |  |  |  |  |
| viii | Language/ cultural differences between physicians and patients. |  |  |  |  |
| ix | Lack of detailed or updated family history |  |  |  |  |
|  |  |  |  |  |  |

**SECTION H. Demographics**

H1. Sex. a. M. b. F

H2. How many years have you been in clinical practice? (excluding internship)

a. 0-9

b. 10-19

c. 20-29

d. 30-39

e. 40 or more

H3. Please indicate your practice setting.

a.Public hospital

b. Private hospital

c. Solo practice

H4. We would like to know if you have a focused area of practice/speciality

a.Internal medicine (no sub-specialisation)

b. Registrar in internal medicine

c. Neurology

d. Cardiology

e. Pulmonology

f. Gastroenterology.

g. Infectious disease

h. Rheumatology

i. Endocrinology

j. Nephrology

H6. Do you have any training in genetics?

a.Yes

b. No

H7. Do you have a special interest in genetics?

a.Yes

b. No

## Appendix C: Permission For Use of Questionnaire.

| \| Carroll, Dr. June <June.Carroll@sinaihealth.ca> \| \| --- \| | Tue, Oct 31, 3:58 PM |  |    |
| --- | --- | --- | --- | --- |
| \| to me   \| \| --- \| | | |  |

Hi Hillary

I am happy to share our study questionnaire. I am out of the country currently and will send it to you when I return at the end of the week.

Regards

June

Sent from my iPhone

‌
